# Global spinal mobility shows no consistent association with trunk strength in children: a school-based cross-sectional study

**DOI:** 10.3389/fspor.2026.1936520

**Published:** 2026-10-26

**Authors:** Julio Martín-Ruiz, Concepción Ros-Ros, Clara Gallego-Cerveró, Laura Ruiz-Sanchis

**Affiliations:** 1 Faculty of Physical Activity and Sports Sciences, Department of Health and Functional Assessment, Catholic University San Vicente Mártir, Valencia, Spain; 2 Faculty of Physical Activity and Sports Sciences, Department of Sports Management and Physical Activity, Catholic University San Vicente Mártir, Valencia, Spain

**Keywords:** biering-sørensen test, deadlift, inertial sensor, low back pain, primary education, secondary education, spinal mobility

## Abstract

**Background:**

Given the high prevalence of low back pain from adolescence, this study examined whether global spinal mobility is associated with trunk strength in healthy schoolchildren.

**Objective:**

To quantify global spinal mobility and movement speed, trunk extensor strength, and associated pain, and to examine their relationships by educational stage and sex.

**Methods:**

In this cross-sectional study, 226 schoolchildren aged 9–16 years completed, in a single Physical Education session, anthropometric measurements, spinal mobility and speed assessments with an inertial sensor, the Biering-Sørensen test, an isometric deadlift with dynamometry, and the Wong-Baker pain scale. *Z*-scores for mobility, speed, and strength were analyzed using two-factor ANOVAs, simple and partial correlations, and linear regression, reporting effect sizes, 95% confidence intervals, and variance inflation factors.

**Results:**

Secondary school students showed greater strength than primary school students (strength index: *p* < 0.001), with no effect of sex; mobility did not differ between stages but was greater in girls (*p* = 0.002), and speed showed a Stage × Sex interaction (*p* = 0.002). Mobility was moderately associated with speed, particularly in primary school, whereas correlations with the composite strength index were non-significant in three of the four subgroups. Secondary school boys showed a small negative association (*r* = −0.25; 95% CI: −0.45 to −0.03; *p* = 0.02), driven mainly by Biering-Sørensen time (*r* = −0.38; *p* < 0.01); after anthropometric adjustment, it was no longer significant for the composite index (partial *r* = −0.22; *p* = 0.06) but persisted for Biering-Sørensen time (partial *r* = −0.31; *p* = 0.01). The mobility–pain relationship was weak, and perceived discomfort was generally low.

**Conclusions:**

Global spinal mobility showed no consistent cross-sectional association with trunk strength in healthy schoolchildren; the only association, observed in secondary school boys, was small and, after adjustment, persisted only for Biering-Sørensen time. Factors not assessed here, such as neural or coordinative aspects, might contribute to this pattern, but this hypothesis requires further investigation. The protocol proved feasible in Physical Education settings, although its screening accuracy has not yet been established.

## 1 Introduction

Good joint health is a key element in addressing any challenges related to physical or athletic activity. The most prestigious guidelines on warm-up routines always include dynamic mobility exercises (1), following the principle that proper mobility must be ensured before placing a load on a segment (2).

When functional deficits prevent the achievement of physiological range of motion, disorders often arise that can become chronic. In the case of the spine, back pain leads to a decrease in quality of life (3), often in the lumbar region, and is so complex to address that, even in the clinical setting, there is a certain lack of motivation regarding the progression of this type of condition (4), which is heterogeneous and in need of highly individualized programs (5). The prevalence of these back conditions is concerning; it is estimated that 80% of the population will experience chronic lower back pain (6). Perhaps even more alarming is its progression; in the child and adolescent population, 38% according to Calvo-Muñoz et al. (7), and more recently, 43.85% according to González-Gálvez et al. (8), before reaching the age of 15, with greater severity in girls due to thoracic abnormalities.

The causes are diverse; however, in adolescents, one of the most influential factors is being overweight or obese (9), in addition to other variables such as anthropometric variables and a sedentary lifestyle (10). In these cases, joint mobility is usually assessed to identify musculoskeletal problems, and typically, they present with more pronounced thoracic kyphosis and reduced range in all flexion movements compared to children without obesity, an aspect that affects not only their posture but also their ability to exert adequate force (11). Mobility deficits are not only due to structural causes; they can also arise from improper postural habits and overexertion (12). As García indicated (13), mobility deficits occur even in athletes who, despite mostly obtaining normal values in spinal alignment tests, present with lumbar or thoracic deviations in approximately 20% of cases.

Puberty is the most favorable period for developing physical activity habits to prevent this situation, and these habits should be continued into adulthood (14). Unfortunately, this is a critical period marked by a significant decline in physical activity (14, 15), among other reasons, due to perceived fatigue and apathy toward enrolling in a medium- to long-term exercise program (16).

Greater practice habits allow for differentiation in the severity of ailments; the more active hours, the less pain is experienced, and vice versa, with trunk flexor-extensor strength and hamstring extensibility being primarily affected (8). Maximum trunk flexion dysfunction is associated with shortening of the lower limb (hamstring muscles) (17), which limits force production (18). In cases where mobility is reduced, trunk muscle strength decreases in 15-year-old students (19), and currently, there are no records for Primary Education students aged 9–12 regarding these variables.

Establishing a correct starting point is essential, and tests capable of assessing the reality of each individual are necessary. Among these is spinal alignment, which, through articular references, can determine whether there are rotations, inclinations, or imbalances between the hemibodies. An example of this is the Langlade test, which is ideal for the school setting as well as for professions with certain physical demands (20), or, more precisely, sensors for obtaining ranges of motion that allow the results to be interpreted and applied in physical conditioning (21). In strength assessment, although dynamic tests are more functional and closer to real-life situations (22), they present lower correlations than those of an isometric nature, which are very close to the actual application of force (23). To perform these exercises, it is advisable to use multi-joint exercises that incorporate coordination and consider the force application vector (horizontal and/or vertical) for a more specific analysis (24). One of the most commonly used tests due to its reproducibility is the Biering-Sørensen paravertebral isometric test (horizontal vector) (25), which uses time to determine the level of stabilization of the spine, establishing, in the case of adults, minimum time thresholds that are considered safe values. It has been used as a standardized test of paravertebral isometric strength in many studies (25, 26) and is more reliable than other similar tests (27). In the case of the vertical force vector, the half squat or squat is one of the most commonly used exercises (28), although its correct execution in isometrics is complicated by the individual's own functional limitations (shortening of the triceps surae, lumbar inversion, poor spinal alignment, etc.) (29). Therefore, alternatives such as the deadlift are more reproducible with an inexperienced sample, as they work the specific agonist musculature (30) and are recognized as preventive exercises widely used for back pain (31, 32).

The ideal framework for putting these principles into practice is Physical Education (PE), in the promotion of physical activity and health within the various existing education laws, with ergonomic education as a fundamental axis (33) and the advantage of being able to apply it continuously and monitored in an educational center.

Therefore, it is important to emphasize the significance of maintaining proper spinal mobility to prevent conditions that may become chronic (34) and ensure the correct and complete application of force in different contexts. The current literature includes some examples of the relationship between these two variables, although referring to adolescents (19), without including Primary Education students. Therefore, the purpose of this project is to calculate the correlation between the range of motion of the spine and the strength of the paravertebral muscles using validated protocols during the growth period and to compare these variables between both educational stages (Primary and Secondary). From a theoretical standpoint, two non-exclusive mechanisms could link spinal mobility to trunk force production: (i) a mechanical pathway, whereby an adequate range of motion allows the trunk extensors to operate closer to their optimal length–tension region and permits the postural adjustments required to transmit force efficiently along the kinetic chain; and (ii) a neuromuscular pathway, whereby the available joint range interacts with proprioceptive feedback, co-contraction strategies and motor control, which are the main determinants of maximal voluntary force during growth. Both mechanisms, however, operate at a regional and task-specific level, so it cannot be assumed *a priori* that the sum of global range of motion adequately reflects them. Accordingly, the present study was designed to quantify cross-sectional associations and not to test predictive or causal relationships. Three hypotheses were formulated: (H1) global spinal mobility would be positively associated with the angular velocity of trunk movements, since both express the available range and its control; (H2) global spinal mobility would show no relevant association (*|r|* < 0.30) with isometric trunk strength once anthropometric characteristics were accounted for; and (H3) the magnitude of these associations would not differ substantially between educational stages or between sexes. Throughout the manuscript, the terms “association” and “relationship” are used to describe cross-sectional covariation, whereas “prediction” and “explanation” are reserved for designs allowing temporal or causal inference and are therefore avoided when interpreting the present findings.

This will allow for a comparison between ages, providing a cross-sectional description of how mobility and strength are expressed at each stage and identify the best strategies for its correction or improvement in the future.

## 2 Materials and methods

### 2.1 Problem statement

To show the correlation between the range of motion of the spine and the capacity to generate voluntary force in the intrinsic musculature of the spine, an analytical, comparative, and cross-sectional study was designed involving students from the Primary (9–11 years) and Secondary (12–16 years) educational stages.

After obtaining authorization from the schools and the participants’ parents (minors), the study measurements were carried out in coordination with the educational center so that they would coincide with the school schedule for Physical Education (PE) classes. The study was conducted in accordance with the guidelines of the Declaration of Helsinki and approved by the Ethics Committee of the Catholic University of Valencia (code: UCV/2024-25/116).

### 2.2 Participants

The participants were Primary Education (96), with 47 boys and 49 girls, and Secondary Education (130), with 79 boys and 51 girls, students from the city of Valencia, Spain. The inclusion criteria were age between 9 and 16 years and regular attendance in PE classes. The only exclusion criteria were recovery from an injury at the time of the study and exemption from participating in PE for other medical reasons.

### 2.3 Procedure

Once it was verified that the students had completed the parental informed consent form, the research was conducted in a single session during the PE class at each school.

First, anthropometric measurements of arm span, biacromial distance, weight, height, and leg length were taken by the principal investigator (ISAK Level I). Arm span was measured using a tape fixed to the wall, recording the distance between the tips of both middle fingers with the shoulders abducted at 90° and elbows extended while the participant maintained an upright posture. For the biacromial distance, the participant was asked to remain in the anatomical position to measure the distance between both acromions (this reference was used as the starting position in two of the three tests performed later).

Height was measured using a stadiometer (Seca 213, Hamburg, Germany) with the participant barefoot and the chin positioned at the Frankfort plane angle. A digital scale (Seca 213, Hamburg, Germany) was used to measure weight, and the measurement was recorded after 2 s in a static position. The last anthropometric test was the measurement of leg length, which was carried out with an anthropometric tape (Lufkin, Apex Tool Group, W606PM, Mexico), with the participant seated, the knee extended, and the foot supported in plantar flexion. The distance between the greater trochanter and foot tip was measured.

Next, the participants performed a standardized warm-up, which consisted of cat-camel exercises (6 reps), bird-dog (6 reps), and front plank (15 s) to promote spinal stabilization, followed by overall joint mobility, and finally, muscle activation exercises: half squat without load (10 reps), shoulder flexion-extension (10 reps), and throws with a 3 kg medicine ball (6 reps). Subsequently, three tests were performed.

### 2.4 Measurement of spinal mobility

The participants were positioned upright with both feet on the ground, spaced at shoulder width. Next, the inertial sensor of the Baiobit device (Rivelo, BTS Engineering Group, Milan, Italy) was placed on the thoracic spine (above the scapula apex). After the sensor was automatically calibrated, each participant was asked to perform three double movements: 1) trunk flexion and extension, 2) right and left trunk rotation, and 3) right and left lateral trunk inclination (Figure 1). All measurements were performed by the same ISAK Level I investigator, previously trained in the standardised use of the device, in order to avoid inter-examiner variability. The Baiobit unit is a wearable inertial measurement unit integrating a triaxial accelerometer, a triaxial gyroscope and a magnetometer, sampling at 200 Hz and transmitting the signal via Bluetooth to the manufacturer's software, which computes angular displacement and peak angular velocity for each movement plane. For every participant the sensor was fixed at the same anatomical landmark by means of a double-sided adhesive patch and an elastic strap to minimise skin artefact, and a static calibration in the anatomical position was performed immediately before testing, defining the zero reference for the three planes. Inertial sensors of this type have shown acceptable concurrent validity and reliability for the assessment of trunk kinematics and control (35), and the thresholds proposed by Koo and Li (36) were adopted to interpret relative reliability.

**Figure 1:**
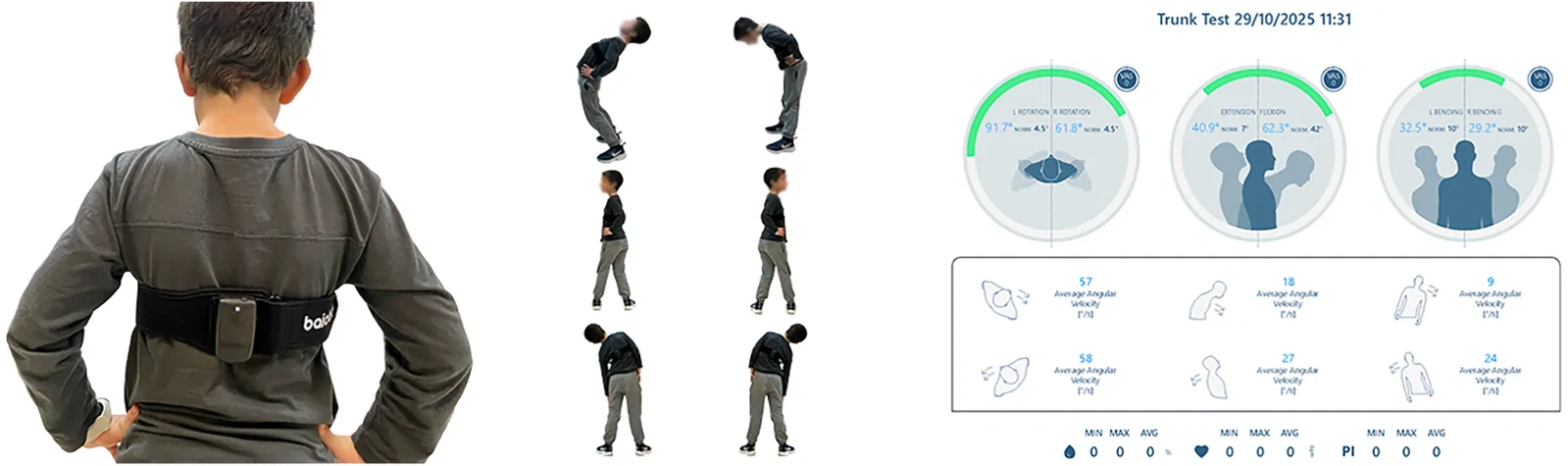
**(A)** Placement of the inertial sensor; **(B)** three double movements; **(C)** mobility report model.

Each double movement was performed twice, with a brief pause in between. At the end of the process, each participant was asked to indicate the level of pain perceived in each isolated movement (flexion, extension, right rotation, left rotation, right flexion, and left flexion) using the Wong-Baker Pediatric Faces scale, from 0 to 10 points (37). Data on amplitude, angular velocity, and pain for each movement were recorded. Verbal instructions were standardised, no manual assistance was provided, and participants were instructed to move to the end of their pain-free range at a self-selected but continuous pace. The highest value of the two trials was retained for each direction, and any trial in which the sensor detached, the feet lost contact with the ground or a compensatory movement of the pelvis was observed was discarded and repeated.

### 2.5 Biering-Sørensen spinal stability test

Next, the participant was asked to lie in a prone position on a stretcher, with the hip positioned at the edge until the end of the stretcher was aligned with their anterior superior iliac crest. The body was secured with straps under the gastrocnemius and gluteal muscles to prevent falling and to ensure stability during the test. A chair was placed in front of the participant so that they could position themselves comfortably and use it at the end of the test. At the investigator's signal, the participant had to keep their spine parallel to the floor by activating the paravertebral muscles, with each hand resting on the opposite shoulder and the cervical spine in a neutral position (Figure 2).

**Figure 2:**
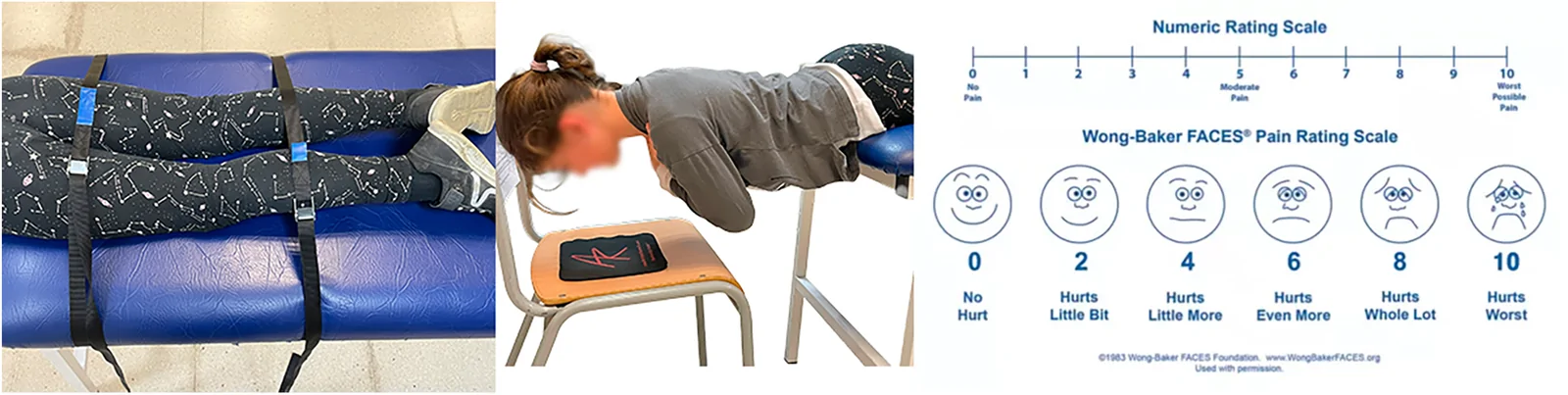
**(A)** Safety grip; **(B)** exercise execution; **(C)** Wong-Baker faces scale.

Each participant performed a familiarization trial and the actual attempt, which ended when there was trembling or movement indicating fatigue (swaying of the trunk, repeated raising or lowering of the shoulders, etc.), and the total duration was recorded in seconds. As in the previous test, perceived pain was recorded at the end of the test. The test was terminated by the same investigator using predefined criteria (loss of the horizontal position of the trunk for more than 5 s despite verbal correction, visible trembling, or voluntary cessation), a procedure that has been shown to be the main source of variability in this test and for which standardisation is recommended (38).

### 2.6 Deadlift

Finally, an isometric deadlift test was performed using a dynamometer (Force Sensor Kit; ChronoJump, Barcelona, Spain). The participants were positioned upright on a steel plate, with their feet set shoulder-width apart. They were provided with a bar for lifting weights, which was attached to chains and a strap on both the plate and the dynamometer.

He was instructed to bend his knees to 110° and keep his spine paravertebrally extended. In this position, the strap was adjusted to eliminate any slack and ensure that the participant's position was not altered during the force-testing. Once this was achieved, the participant was given two verbal cues: the first to pull progressively, and the second to pull with maximum effort. This procedure was used to avoid abnormal spikes in the recorded signals (Figure 3).

**Figure 3:**
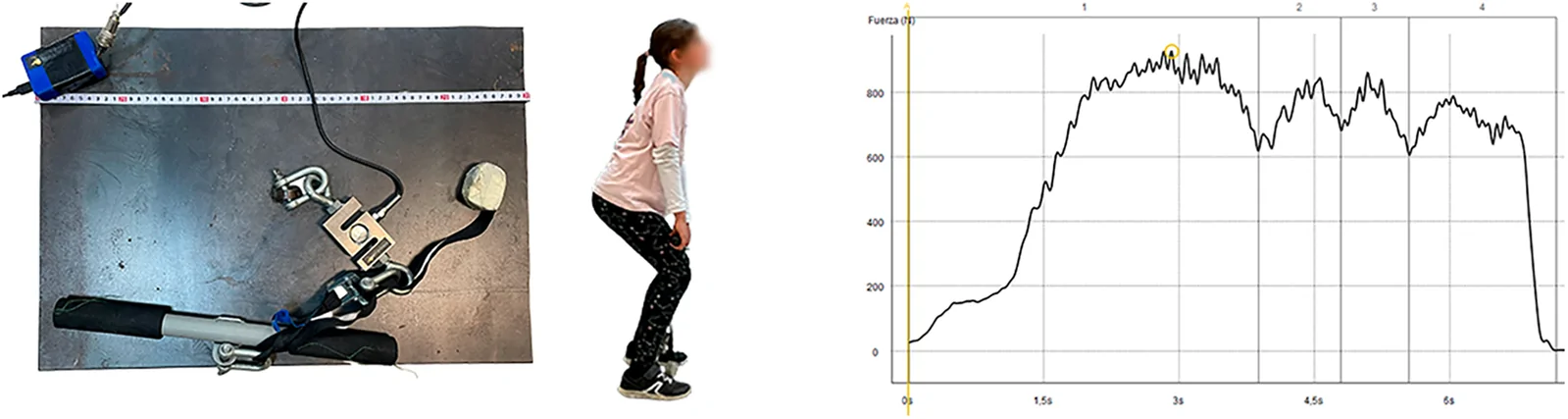
**(A)** Dynamometer, grip, and plate; **(B)** deadlift exercise; **(C)** registration signal.

Each participant performed two attempts with a 2-min pause to prevent fatigue and ensure the achievement of maximum possible strength, compensating for their understandable inexperience with this type of maximal test. The value was recorded in Newtons, with the analysis utilizing the highest force attempt and the recording of perceived pain, as in the previous two tests. Relative reliability between the two deadlift attempts in the present sample was excellent, with an intraclass correlation coefficient ICC(3,1) = 0.948 (95% CI: 0.932–0.959), a standard error of measurement of 93.8 N, a coefficient of variation of 13.3% and a minimal detectable change (MDC95) of 260.1 N, interpreted according to the reliability thresholds described above.

### 2.7 Statistical analysis

Statistical analyses were performed using R software (39). A significance level of *p* < 0.05 was set, and when relevant, effect sizes were reported, and the corresponding confidence intervals were considered.

First, a descriptive analysis was conducted for all anthropometric variables (age, weight, height, body mass index, arm span, and leg length), spinal mobility, movement speed, muscle strength, and pain level. For each combination of educational stage (Primary, Secondary) and sex (boys and girls), means, standard deviations, minimums, maximums, and sample sizes were calculated.

To explore global differences between stage and sex in the main continuous variables (maximal strength, Biering-Sørensen time, mobility indexes, and velocity), two-factor analyses of variance (ANOVA Stage × Sex) were used, using Type III sums of squares and reporting the F statistic, the corresponding *p*-value and partial eta squared (*η_p_*^2^) for the main effects of stage and sex and for the Stage × Sex interaction.

Composite indices were defined based on the spinal mobility measurements. The global mobility index (Mobility_index) was constructed as the sum of the range of motion in flexion, extension, right and left rotation, and right and left lateral flexion. Similarly, a global speed index (Speed_index) was obtained by adding the angular velocities recorded for all six movements. For strength, the time in the Biering-Sørensen test and the maximum isometric force recorded in the deadlift (*N*) were used. Each variable was first standardized as a *z*-score using the mean and standard deviation of the whole sample (*n* = 226), and the global strength index was then calculated as the unweighted arithmetic mean of the two *z*-scores: Strength_index_*z* = (*z*_Biering-Sørensen + *z*_deadlift)/2. The index was not re-standardized, so its standard deviation is lower than 1 (Table 1). Additionally, the mobility and speed indices were transformed into *z*-scores (Mobility_index_*z*, Speed_index_*z*) to facilitate comparison and interpretation in standard deviation units. The construction of these composite indices followed a dimension-reduction criterion: analysing the six movement directions and the two strength tests separately would have multiplied the number of contrasts and inflated the type I error rate, whereas the indices summarise the total range of motion available to the trunk and the overall level of trunk force. Their internal consistency was examined before use: Cronbach's alpha was 0.80 for the velocity index and 0.73 for the mobility index, and the correlation between the two components of the strength index was *r* = −0.034 (*p* = 0.610). These values indicate that both indices must be interpreted as descriptive summaries of global performance and not as indicators of a single latent construct, since the Biering-Sørensen test (isometric endurance of the extensors) and the isometric deadlift (maximal multi-joint force) capture different neuromuscular qualities. The purpose of combining them was not to measure a single latent trait, but to obtain one pre-specified summary outcome of overall trunk-extensor and posterior-chain strength for the primary analysis, thereby limiting the number of contrasts. The two tests were chosen because they are complementary: the Biering-Sørensen test assesses the isometric endurance of the trunk extensors against body weight along a horizontal force vector, whereas the isometric deadlift assesses the maximal force production of the trunk extensors and posterior chain along a vertical force vector (see Introduction). Equal weights were assigned because there was no *a priori* reason to consider one test more relevant than the other. Because the two components were practically uncorrelated, a given value of the index can result from different combinations of endurance and maximal force, and opposite associations of mobility with each component may partly cancel each other out in the composite. For this reason, each mobility direction and each strength test were additionally analysed separately as a sensitivity analysis, and these results are reported alongside those obtained with the composite indices; when the composite index and its components diverged, interpretation was based on the components. Prior to the analyses, the database underwent a quality-control review in which every variable was inspected for values outside physiologically plausible ranges and cross-checked against the original sensor exports. This procedure identified two data-entry errors, which were corrected against the source records: one range-of-motion value for left rotation and eight angular-velocity values for left lateral flexion whose sign had been inverted during transcription. No participant was excluded on this basis.

**Table 1:** Descriptive statistics of mobility, speed, and strength indices by stage and sex.

Stage	Sex	*N*	Mobility raw	Mobility *z*	Speed raw	Speed *z*	Strength *z*
Primary	Male	47	399.19 ± 56.79	−0.17 ± 1.01	207.13 ± 66.13	−0.20 ± 0.91	−0.40 ± 0.63
Primary	Female	49	425.22 ± 51.59	0.30 ± 0.92	254.53 ± 77.49	0.45 ± 1.07	−0.28 ± 0.65
Secondary	Male	79	397.70 ± 53.15	−0.19 ± 0.95	219.65 ± 73.53	−0.03 ± 1.01	0.27 ± 0.65
Secondary	Female	51	418.12 ± 60.13	0.17 ± 1.07	207.29 ± 64.01	−0.20 ± 0.88	0.22 ± 0.60

Different approaches have been used to analyze the relationship between spinal mobility and physical performance. First, Pearson's correlations were calculated between mobility and speed indices, as well as between mobility and strength variables (Biering-Sørensen time, maximum deadlift strength, and Strength_index_*z*), stratified by educational stage and sex. To control for the possible influence of anthropometric characteristics, partial Pearson correlations were calculated using the pcor function (40), adjusting for age, weight, height, body mass index, arm span, and leg length.

In addition to correlations, linear regression models were used to further examine the relationship between mobility and muscle strength. Simple models were estimated with Strength_index_*z* as the dependent variable and Mobility_index_*z* as the predictor, stratified by stage and sex. Subsequently, multiple models were adjusted by adding covariates such as age, weight, height, body mass index, arm span, and leg length. From each model, the regression coefficient for mobility (beta), standard error, *t*-statistic and *p-value* were extracted. Covariates were selected *a priori*, before inspecting the results and without stepwise procedures, on conceptual rather than statistical grounds (41): age, weight, height, body mass index, arm span and leg length are markers of biological growth and body size that simultaneously condition the range of motion recorded by the sensor and the capacity to produce force, and therefore act as potential confounders of the mobility–strength relationship. They were retained together in the primary adjusted models so that the adjustment for body size and growth would be as complete as possible and consistent with the partial correlations (the test of a partial correlation is mathematically equivalent to the test of the corresponding coefficient in the adjusted regression model). We acknowledge, however, that these covariates are strongly interrelated, since body mass index is derived from weight and height, and arm span and leg length are closely related to height. Model diagnostics were examined in every case: variance inflation factors (VIF) were computed for all predictors and the explanatory power of the models was quantified by means of *R*^2^ and adjusted *R*^2^. Given that weight, height and body mass index are mathematically interdependent, a sensitivity model with a reduced set of covariates (age, body mass index and leg length) was additionally estimated in order to confirm that the mobility estimate was not distorted by collinearity among the anthropometric covariates. The covariates of the reduced model were chosen to retain one non-redundant indicator of each dimension: age (growth and maturation), body mass index (body mass relative to stature) and leg length (segment length, which affects the lever arms in both strength tests). Multicollinearity does not bias ordinary least squares estimates, but it inflates their variance; because the mobility index showed low VIF values, its standard error was not inflated, whereas those of the anthropometric covariates were. Therefore, only the mobility coefficient was interpreted, the coefficients of the individual anthropometric covariates were not interpreted, and the stability of the mobility estimate across the fully adjusted and reduced models was used as the criterion of robustness. Given the limited number of observations per predictor in the stratified fully adjusted models (approximately 7–11), the adjusted estimates were interpreted with caution.

The relationship between mobility and pain was analyzed using the mean pain index associated with spinal movements (Pain_mobility_index), which was calculated as the average pain score during flexion, extension, rotation, and lateral bending. Pearson correlations were obtained between the pain index, mobility index, and speed index, stratified by stage and sex. These correlations, along with their *p*-values, are presented in tables that summarize the association between mobility, functional performance, and perceived pain. In all cases, Pearson's correlation tests were used because the variables were continuous and approximately normally distributed, and partial versions were applied when it was necessary to control for anthropometric covariates. No correction for multiple comparisons was applied, as the contrasts derive from a limited set of pre-specified hypotheses and not from an exploratory search of the data; all *p*-values reported in the text and in the tables are therefore unadjusted. Consistent with this decision, and given the exploratory nature of the stratified analyses, interpretation is based primarily on effect sizes and their 95% confidence intervals rather than on statistical significance alone.

*Post-hoc* power analysis (G*Power 3.1) indicated a statistical power of 0.95 to detect *r* = 0.30 with *n* = 226, but the power was reduced to 0.60 in stratified analyses (*n* ∼ 50 per subgroup). Consequently, the stratified analyses must be regarded as exploratory. Estimates are therefore reported together with their 95% confidence intervals and the corresponding effect sizes, and the absence of statistical significance within a subgroup is interpreted as an imprecise estimate compatible with a small effect, and not as evidence of the absence of a relationship.

## 3 Results

### 3.1 Sample characteristics

The sample consisted of Primary and Secondary Education students stratified by sex. The anthropometric characteristics of the participants are shown in Table 2.

**Table 2:** Descriptive anthropometric characteristics of the sample by stage and sex.

Stage	Sex	*N*	Age	Weight	Height	BMI	Arm span	Biacromial distance	Leg length
Primary	Male	47	10.17 ± 0.92	41.29 ± 10.18	143.37 ± 7.78	19.93 ± 3.79	142.12 ± 9.20	34.19 ± 2.60	89.66 ± 6.17
Primary	Female	49	10.24 ± 0.90	40.62 ± 9.41	145.17 ± 8.43	19.10 ± 3.24	142.67 ± 9.11	33.51 ± 2.29	90.37 ± 5.58
Secondary	Male	79	13.57 ± 1.07	58.76 ± 13.26	166.63 ± 9.70	21.01 ± 3.67	164.77 ± 20.15	39.11 ± 3.10	100.72 ± 7.12
Secondary	Female	51	13.73 ± 1.04	53.95 ± 10.03	159.55 ± 7.05	21.13 ± 3.28	158.43 ± 8.63	37.55 ± 2.27	97.12 ± 5.26

BMI, body mass index.

Table 3 presents the records of spinal mobility in the sample.

**Table 3:** Descriptive data on spinal range of motion (ROM) by stage and sex.

Stage	Sex	*N*	Trunk flexion	Trunk extension	Right rotation	Left rotation	Right lateral flexion	Left lateral flexion
Primary	Male	47	95.34 ± 17.88	41.55 ± 11.26	85.85 ± 16.70	85.67 ± 14.54	43.11 ± 11.82	47.67 ± 19.96
Primary	Female	49	102.91 ± 19.18	48.83 ± 13.22	90.60 ± 13.53	90.89 ± 13.76	44.66 ± 10.07	47.33 ± 12.79
Secondary	Male	79	96.09 ± 17.23	44.29 ± 11.22	88.86 ± 15.20	88.01 ± 12.70	40.83 ± 9.02	39.62 ± 13.34
Secondary	Female	51	100.77 ± 21.31	50.01 ± 12.82	90.19 ± 12.56	91.09 ± 15.50	43.25 ± 9.19	42.81 ± 11.18

The data on the angular velocities of the six movements of the spine are listed in Table 4.

**Table 4:** Descriptive data of angular velocity (°/s) by stage and sex.

Stage	Sex	*N*	Trunk flexion speed	Trunk extension speed	Right rotation speed	Left rotation speed	Right lateral flexion speed	Left lateral flexion speed
Primary	Male	47	22.02 ± 12.06	38.89 ± 20.88	51.15 ± 18.69	49.98 ± 20.56	21.23 ± 9.46	23.85 ± 14.92
Primary	Female	49	32.22 ± 17.07	48.73 ± 27.27	57.69 ± 20.79	54.98 ± 18.31	31.76 ± 13.65	29.14 ± 13.63
Secondary	Male	79	25.62 ± 12.62	41.77 ± 21.04	55.22 ± 22.71	52.73 ± 19.06	21.96 ± 9.42	22.34 ± 12.45
Secondary	Female	51	25.88 ± 11.73	41.96 ± 18.28	52.22 ± 18.93	44.80 ± 13.40	22.04 ± 12.00	20.39 ± 9.42

Table 5 shows the average times of the Biering-Sørensen test and the strength of isometric deadlift.

**Table 5:** Descriptive data of the time in the Biering-Sørensen test and strength in the deadlift.

Stage	Sex	*N*	Biering-Sørensen (s)	Best deadlift (N)
Primary	Male	47	39.44 ± 16.09	506.90 ± 261.57
Primary	Female	49	43.06 ± 14.42	506.53 ± 335.85
Secondary	Male	79	46.23 ± 17.35	900.90 ± 525.43
Secondary	Female	51	50.16 ± 13.53	746.14 ± 316.39

N., Newtons.

To compare the overall differences between stage and sex, a two-factor ANOVA (Stage × Sex) with associated *F* and *p*-values was applied to each continuous variable. Secondary school students showed greater height and weight (Table 2) and higher performance in both strength tests than primary school students [Biering-Sørensen: *F*(1, 222) = 10.62, *p* = 0.001, *η_p_*^2^ = 0.05; deadlift: *F*(1, 222) = 34.34, *p* < 0.001, *η_p_*^2^ = 0.13], with no significant effect of sex (*p* = 0.078 and *p* = 0.153, respectively) or Stage × Sex interaction (*p* = 0.942 and *p* = 0.155). Total spinal mobility did not differ between stages [*F*(1, 222) = 0.33, *p* = 0.568, *η_p_*^2^ < 0.01] but was greater in girls than in boys [*F*(1, 222) = 9.56, *p* = 0.002, *η_p_*^2^ = 0.04], with no interaction (*p* = 0.709). For total movement speed, the main effects of stage (*p* = 0.073) and sex (*p* = 0.070) were not significant, but a significant Stage × Sex interaction was observed [*F*(1, 222) = 9.61, *p* = 0.002, *η_p_*^2^ = 0.04]: girls were faster than boys in Primary school, whereas this difference was not observed in Secondary school.

### 3.2 Mobility, speed, and strength indexes

To facilitate global interpretation, standardized indices were constructed for the entire sample (Table 1).

Mobility_index_*z*: *z*-score of the sum of the ranges of trunk movement (flexion, extension, right and left rotation, and right and left lateral flexion).

Speed_index_*z*: *z*-score of the sum of the speeds of the six movements.

Strength_index_*z*: average of the *z*-scores for the time in the Biering-Sørensen test and maximum deadlift strength.

ANOVA (Stage × Sex) was performed on *z*-scores to assess the effects of stage and sex. By construction, the mean of each index in the whole sample was 0. Secondary school students showed higher values in the strength index (Strength_index_*z*) than Primary school students [*F*(1, 222) = 45.88, *p* < 0.001, *η_p_*^2^ = 0.17], with no significant effect of sex (*p* = 0.744) or Stage × Sex interaction (*p* = 0.328), consistent with higher strength levels at older ages, although the cross-sectional design does not allow developmental trajectories to be inferred. Because the *z*-transformation is linear, the ANOVA results for the mobility and speed indices were identical to those reported above for total mobility and total speed.

### 3.3 Relationship between mobility, speed, and strength of the spine

In Table 6, the Pearson correlations are highlighted between the global mobility index and speed_index, time in Biering-Sørensen, deadlift strength, and Strength_index_*z*.

**Table 6:** Correlations between the spinal mobility index and speed and strength outcomes by stage and sex.

Stage sex	Outcome	*r* simple	*p* simple	*r* partial	*p* partial	Cohen d Pearson
Primary Male	Speed index	0.44	0.00	0.42	0.01	0.969
Primary Male	Biering-Sorensen	−0.15	0.32	0.08	0.64	−0.298
Primary Male	Best deadlift	−0.30	0.04	−0.05	0.77	−0.628
Primary Male	Strength index	−0.26	0.07	0.04	0.81	−0.545
Primary Female	Speed index	0.45	0.00	0.45	0.00	1.005
Primary Female	Biering-Sorensen	0.11	0.45	0.10	0.53	0.220
Primary Female	Best deadlift	−0.26	0.07	−0.17	0.27	−0.548
Primary Female	Strength index	−0.08	0.57	−0.03	0.85	−0.166
Secondary Male	Speed index	0.13	0.26	0.30	0.01	0.258
Secondary Male	Biering-Sorensen	−0.38	0.00	−0.31	0.01	−0.826
Secondary Male	Best deadlift	0.07	0.55	0.00	1.00	0.138
Secondary Male	Strength index	−0.25	0.02	−0.22	0.06	−0.524
Secondary Female	Speed index	0.15	0.28	0.38	0.01	0.312
Secondary Female	Biering-Sorensen	0.09	0.53	0.24	0.11	0.180
Secondary Female	Best deadlift	0.18	0.20	0.13	0.38	0.369
Secondary Female	Strength index	0.17	0.22	0.24	0.11	0.354

*r*, Pearson correlation coefficient; *r* partial, partial correlation adjusted for age, weight, height, body mass index, arm span and leg length; Cohen *d*, effect size derived from *r*. All *p*-values are unadjusted; no correction for multiple comparisons was applied.

The correlations between global spine mobility and the speed index were low or moderate. In Primary school, the mobility–speed relationship was higher and significant in both boys (*r* = 0.44; *p* < 0.01) and girls (*r* = 0.45; *p* < 0.01); in Secondary school the simple associations were low and non-significant (boys *r* = 0.13; girls *r* = 0.15), although they reached significance after adjustment for anthropometric characteristics (partial *r* = 0.30 and 0.38, respectively; *p* = 0.01). However, the correlations between mobility and strength (Biering-Sørensen, deadlift, and Strength_index) were weak, and reached statistical significance in only two cases: in Primary school boys, a small negative correlation with deadlift strength (*r* = −0.30; *p* = 0.04) that disappeared after adjustment for anthropometric characteristics (partial *r* = −0.05; *p* = 0.77); and in Secondary school boys, a negative correlation with Biering-Sørensen time (*r* = −0.38; *p* < 0.01), which was attenuated but persisted after adjustment (partial *r* = −0.31; *p* = 0.01) and was reflected in a small negative correlation with the Strength_index (*r* = −0.25; *p* = 0.02). This suggests that, in this sample, the variation in strength was largely unrelated to differences in total spinal mobility.

### 3.4 Relationship between mobility and strength with standardized indices

Table 7 shows the relationships between the global mobility *z*-score index and the global strength *z*-score index, calculated using bilateral and partial Pearson correlations.

**Table 7:** Pearson and partial correlations between *z*-standardized spinal mobility and strength indices according to stage and sex.

Stage	Sex	*N*	*r* Pearson	*p* Pearson	*r* partial	*p* partial	Cohen *d* Pearson
Primary	Male	47	−0.26	0.07	0.04	0.81	−0.545
Primary	Female	49	−0.08	0.57	−0.03	0.85	−0.166
Secondary	Male	79	−0.25	0.02	−0.22	0.06	−0.524
Secondary	Female	51	0.17	0.22	0.24	0.11	0.354

*r*, Pearson correlation coefficient; *r* partial, partial correlation adjusted for age, weight, height, body mass index, arm span and leg length; Cohen *d*, effect size derived from *r*. All *p*-values are unadjusted; no correction for multiple comparisons was applied.

In three of the four groups, the correlations between the *z* mobility index and the *z* strength index were low and non-significant (*p* > 0.05), whereas Secondary school boys showed a small negative association (*r* = −0.25; *p* = 0.02). The partial correlations, after adjusting for anthropometry, did not reach statistical significance in any subgroup, including Secondary school boys (partial *r* = −0.22; *p* = 0.06). These results indicate, as in the previous case, that overall spinal mobility is not consistently associated with strength performance in children, and that the only association detected with the composite index did not persist once body size was controlled for, although in this subgroup the association with Biering-Sørensen time did persist (Table 6). To make the interpretation transparent, the point estimates and their 95% confidence intervals were as follows: Primary boys *r* = −0.263 (95% CI: −0.51 to 0.03; *n* = 47), Primary girls *r* = −0.083 (95% CI: −0.36 to 0.20; *n* = 49), Secondary boys *r* = −0.254 (95% CI: −0.45 to −0.03; *n* = 79) and Secondary girls *r* = 0.174 (95% CI: −0.11 to 0.43; *n* = 51). Three of the four intervals include zero and are compatible with small effects in either direction; only in Secondary school boys does the interval exclude zero, although its upper limit (−0.03) is also compatible with a negligible effect. The sensitivity analysis by individual dimensions in the whole sample (*n* = 226) was consistent: when each movement direction was correlated separately with each strength test, all coefficients were small (*|r|* ≤ 0.28), the largest being that between left lateral flexion and Biering-Sørensen time (*r* = −0.282; *p* < 0.001), while the remaining statistically significant associations were of negligible magnitude (*|r|* ≤ 0.16). For the sample as a whole, global mobility was associated neither with Biering-Sørensen time (*r* = −0.118; *p* = 0.078) nor with maximal deadlift strength (*r* = −0.060; *p* = 0.372).

Table 8 presents the ordinary linear regressions with *β* coefficient estimates by stage and sex.

**Table 8:** Linear regression models of the strength index on the mobility index by stage and sex.

Stage	Sex	*N*	*β* simple	se simple	*t* simple	*p* simple	*β* adj	se adj	*t* adj	*p* adj
Primary	Male	47	−0.16	0.09	−1.83	0.07	0.02	0.10	0.24	0.81
Primary	Female	49	−0.06	0.10	−0.57	0.57	−0.02	0.11	−0.19	0.85
Secondary	Male	79	−0.17	0.08	−2.30	0.02	−0.17	0.09	−1.90	0.06
Secondary	Female	51	0.10	0.08	1.24	0.22	0.15	0.09	1.66	0.11

*β*, standardized coefficient representing the change in the strength index per 1 *SD* increase in the mobility index; se, standard error; *t*, *t*-statistic; adj, model adjusted for age, weight, height, body mass index, arm span and leg length. All *p*-values are unadjusted; no correction for multiple comparisons was applied.

The mobility index reached statistical significance only in the simple model for Secondary school boys (*β* = −0.17; *p* = 0.02); in the remaining subgroups, and in all adjusted models, the coefficients were non-significant (*p* > 0.05). Among Secondary school girls, a trend toward a positive association was observed in the adjusted model (*β* = 0.15; *p* = 0.11), while among Secondary school boys the negative coefficient retained its magnitude but lost significance after adjustment (*β* = −0.17; *p* = 0.06). Overall, the contribution of spinal mobility to the variability in strength was limited. Regarding explanatory power, the simple models accounted for between 0.7% and 6.9% of the variance in the strength index (*R*^2^ = 0.007–0.069; adjusted *R*^2^ ≤ 0.052), and the fully adjusted models for between 8.4% and 33.2% (*R*^2^ = 0.084–0.332; adjusted *R*^2^ = −0.031–0.212), an increase attributable to the anthropometric covariates and not to mobility. Collinearity diagnostics showed that, in the fully adjusted models, weight, height and body mass index presented very high variance inflation factors (VIF > 30 in every subgroup, up to 209.7), as expected from their mathematical interdependence, whereas the mobility index remained unaffected (VIF = 1.1–1.5), indicating that its estimate was stable. In the sensitivity model with a reduced set of covariates (age, body mass index and leg length), all VIF values were below 1.9 and the pattern remained unchanged: *β* = −0.057 (*p* = 0.566) in Primary boys, *β* = −0.039 (*p* = 0.705) in Primary girls, *β* = −0.156 (*p* = 0.077) in Secondary boys and *β* = 0.143 (*p* = 0.116) in Secondary girls (*R*^2^ = 0.068–0.208). Because the mobility estimate was similar in the fully adjusted and reduced models, the conclusions do not depend on the choice of covariate set; nevertheless, given the small subgroup sizes relative to the number of covariates, the adjusted estimates should be interpreted with caution.

In Figure 4, the regression lines corresponding to simple linear models (strength as the dependent variable and mobility as the predictor) by stage and sex can be observed, equivalent to the beta_simple coefficients presented in Table 8.

**Figure 4:**
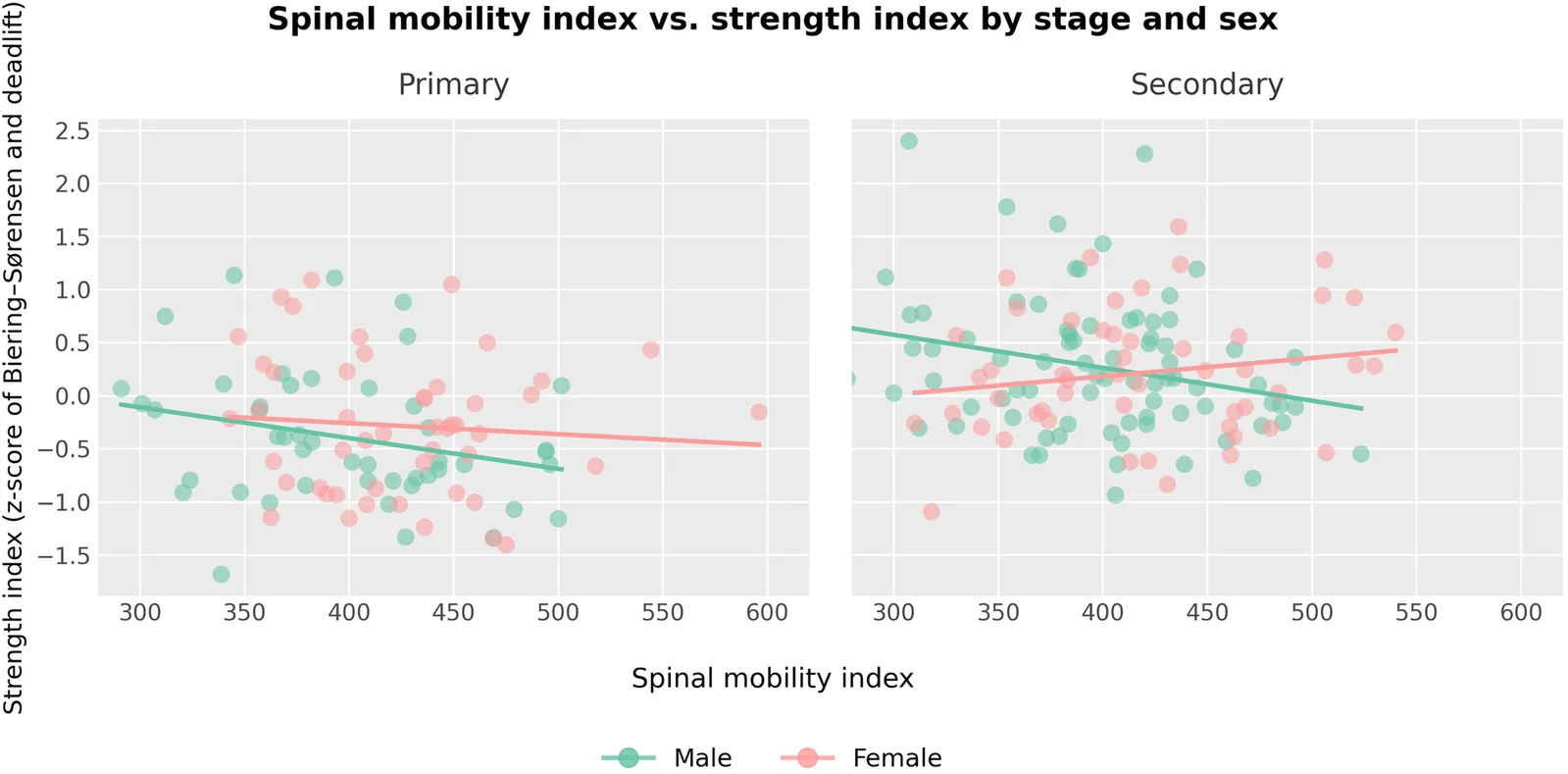
Relationship between spinal mobility index and strength index by stage and sex.

The absence of a trend between global spinal mobility and strength is confirmed by the scatter plots, which are dispersed, the slopes of the regression lines are almost horizontal in most panels, and there are no visually significant differences between the sexes. This is consistent with the numerical results obtained from the correlation and regression models.

### 3.5 Relationship between mobility, speed, and pain

Table 9 presents the results of the development of a pain index associated with spinal movements and its analysis using two-tailed Pearson correlations between pairs of indices.

**Table 9:** Correlations between spinal mobility, speed, and pain indices by stage and sex.

Stage	Sex	*N*	*r* mobility speed	*p* mobility speed	*r* mobility pain	*p* mobility pain
Primary	Male	47	0.44	0.00	0.05	0.75
Primary	Female	49	0.45	0.00	−0.28	0.05
Secondary	Male	79	0.13	0.26	−0.01	0.96
Secondary	Female	51	0.15	0.28	−0.10	0.48

*r*, Pearson correlation coefficient. All *p*-values are unadjusted; no correction for multiple comparisons was applied.

Spinal mobility was positively and moderately associated with movement speed, especially in primary school (significant in both boys and girls), whereas in secondary school the simple correlations were low and non-significant (Table 9). The relationship between mobility and pain was weak and, in most cases, not significant. In Primary school girls, there was a small, borderline negative correlation (*r* = −0.28; *p* = 0.05), indicating that higher mobility values tended to be associated with lower average pain. Overall, the data do not support a direct relationship between greater overall spinal mobility and increased pain in healthy children at these ages.

## 4 Discussion

Spinal health is essential at an early age because it is still developing and is in constant interaction with soft tissues that are subject to continuous variations in amplitude and tension (9, 42). Growth involves going through logical anabolic phases, in which the ability to generate strength increases, requiring a balance between the tension and relaxation of different muscle and joint structures (43). This study provides an analysis of a sensitive population aged 9–16 years with unprecedented objective measures of spinal mobility, speed, and strength combined in a single physical fitness protocol along with perceived pain.

In this cross-sectional design, global spinal mobility showed no consistent association with trunk strength in children, and the only significant association with the composite strength index, found in secondary school boys, was small, negative and did not persist after adjusting for body size, although the association with Biering-Sørensen time did. Since neural, coordinative and technical factors were not assessed in the present study, these data cannot establish what determines trunk strength in this population. It is possible that such factors (44), probably perceptual-motor and coordinative, play a more relevant role than global range of motion, as might specific profiles of localized hypermobility or differences in execution technique; however, these explanations remain hypotheses that require studies in which these variables are measured directly.

Several reasons may explain why global spinal mobility was not consistently associated with trunk strength. The explanations proposed below are based on the literature and were not directly tested in this study, so they should be considered possible interpretations rather than conclusions supported by the present data. First, flexibility and strength are relatively independent physical qualities during growth: the available range of motion depends largely on the mechanical properties of connective tissue and on stretch tolerance, whereas maximal voluntary force depends on muscle cross-sectional area and, above all, on neural factors such as motor unit recruitment, firing frequency and intermuscular coordination, which are the main determinants of strength gains in children and adolescents (45–47). Second, any such association is likely to be regional and task-specific rather than global: the paravertebral muscles work within a narrow range around the neutral position in both strength tests, so the total range of motion available adds little information about the capacity to stabilise the trunk, and only a deficit severe enough to prevent the adoption of the test position would be expected to limit force production. Third, in a healthy school-aged sample the range of both variables is restricted and there are no extreme values of hypomobility or weakness, which attenuates any linear association; such an association might emerge in clinical populations, in whom structural and functional alterations of the back muscles have been documented (48). Finally, motor control and execution technique, which were not quantified in this study, may act as intermediate variables masking the mobility–strength relationship, since two participants with the same range of motion may express very different force levels depending on their stabilisation strategy.

The small negative association observed in secondary school boys deserves specific comment. It was concentrated in the Biering-Sørensen test (*r* = −0.38; *p* < 0.01) rather than in maximal deadlift force (*r* = 0.07; *p* = 0.55), which suggests that it relates to isometric endurance of the trunk extensors rather than to maximal force production. This divergence also illustrates why the composite Strength Index must be interpreted together with its components: because the two tests capture different qualities, the index summarises overall trunk-strength performance but may dilute or mask component-specific associations. Two non-exclusive explanations are plausible. On the one hand, this is the subgroup with the greatest anthropometric heterogeneity, as it spans the pubertal growth spurt: greater body size increases the external torque that must be sustained in the Biering-Sørensen position and is simultaneously associated with longer segment lengths and larger absolute ranges of motion, so part of the association may reflect body size rather than mobility itself. This interpretation is consistent with the attenuation of the coefficients after adjusting for anthropometric covariates (composite index: partial *r* = −0.22; *p* = 0.06; Biering-Sørensen time: partial *r* = −0.31; *p* = 0.01), although the persistence of the latter indicates that body size does not fully account for the association. On the other hand, a greater available range of motion, in the absence of a proportional stabilisation capacity, could reduce the efficiency of the isometric holding strategy. Since this association appeared in only one of the four subgroups, was only partially attenuated after adjustment and, for the composite index, rests on a confidence interval whose upper limit is compatible with a negligible effect, it should be treated as a hypothesis to be tested in future studies rather than as an established finding.

The analysis of pain has played an essential role in monitoring, and must always be considered, given its constant presence during adulthood (49–51). Its relationship with other variables derived from physical conditions is highly relevant in PE and fulfills a clear preventive role. In this regard, the work adds to the postural fitness line of screening protocols in schoolchildren, such as those by Martínez-Romero et al. (52), who aimed to improve strength levels, and others who emphasized the importance of mobility at an early age (53), which aligns with the purpose of this study.

The speed of each movement showed a moderate association with mobility, particularly in the primary group. This is possibly a reflection of the relationship between the available range of motion and motor control in the early stages, in line with other studies that link mobility, flexibility, and trunk control with functional performance (54, 55) and risk of pain. These relationships are multifactorial and non-linear (56); therefore, they cannot be attributed to a single factor.

Owing to its cross-sectional nature, the study captures the association between mobility and both strength tests at a single point in time, so regional movement patterns may not be reflected (57). Technical aspects not captured in this study could become more evident over time and modify this association, which can only be examined with longitudinal designs. In addition, this component has been studied more extensively in specific adult populations (57, 58) and healthy students with lower incidence rates.

The protocol offers the possibility of being applied in a single PE session, taking into account anthropometric, joint and muscle physiology variables, along with perceptual ones, all using accessible and easily reproducible tests. This is of great help to PE teachers (59) as it provides a useful resource for creating early detection programs and postural education. The ways of integrating it into programming units could be very broad: preventive analytical exercises during warm-up phases, as in previous studies (23, 60, 61), as the final part of units in which strength is the main focus, and even integrated into standard test batteries as an alternative to questionable tests that focus on spinal stability (the Biering-Sørensen test could replace the abdominal test in the Eurofit Battery). It must nevertheless be stressed that this study examined associations between functional measures and did not evaluate diagnostic accuracy in terms of sensitivity, specificity or ROC curves against any clinical reference standard. The protocol should therefore be described as a feasible and reproducible approach for assessing physical function in the school setting, and not as a validated screening instrument; establishing its screening properties would require prospective designs with clinically relevant outcomes and analyses of diagnostic accuracy, in line with the caveats raised concerning the predictive capacity of screening tests in the field of physical activity and health (62).

The differences between sexes have not been significant, especially in Primary Education, confirming what has been found in other recent studies (63, 64), although descriptively the difference in deadlift strength between stages was larger in boys, in line with the expected increase in strength among boys during puberty (secondary school) reported in various studies (65, 66).

Regardless of the results obtained, the usual recommendations for extensive muscle stimulation work (67), preferably isometric exercises for the paravertebral muscles, remain highly recommended in the literature. In addition, closer monitoring of both postural habits—which add to risk factors (sedentary lifestyle, screen time, etc.)—and the evolution of lumbar strength in girls, due to the higher prevalence of pain in adulthood, is important (68), and would be very relevant in both school and work contexts.

The study presents some limitations, such as the possibility of expanding the tests carried out with the Baiobit sensor, for example, by analyzing the cervical spine of participants. However, a greater number of strength assessment tests, such as dynamic tests with a linear encoder, could be used to establish an accurate force-velocity profile. Additionally, longitudinal interventions could be conducted to assess the acute effect at each moment and the change in mobility and strength variables, with the possibility of including other variables such as academic performance. Beyond these aspects, the study has limitations that condition the interpretation of the findings. Its cross-sectional design allows covariation to be observed at a single point in time, but not temporal or causal relationships to be established; the results should therefore not be read as evidence that mobility does not influence the development of trunk strength, but as the absence of a significant cross-sectional association in this sample. In addition, the composite indices summarise heterogeneous dimensions and, as reflected in their internal consistency, do not represent a single latent construct, so their interpretation should be complemented by the analysis of the individual dimensions. In particular, the Strength Index combines two practically uncorrelated tests (*r* = −0.034), so it should be read as a descriptive summary of overall trunk-strength performance rather than as a measure of a single strength capacity. Likewise, the anthropometric covariates were strongly interrelated, which produced high variance inflation factors in the fully adjusted models; although the mobility estimate was stable across the fully adjusted and reduced models, the small subgroup sizes relative to the number of covariates limit the precision of the adjusted estimates. The stratified analyses (n ≈ 47–79 per subgroup) had limited statistical power to detect small effects, and their estimates, although accompanied by confidence intervals, remain imprecise. Finally, biological maturation status (for example, through peak height velocity) and the level of habitual physical activity were not assessed, both of which could modulate the relationship studied; longitudinal designs with repeated measurements throughout the growth spurt would be required to determine whether mobility precedes, accompanies or is independent of the development of trunk strength.

In this regard, it would be interesting to assess these aspects under different conditions, such as fatigue, to determine the degree of variation compared with neutral situations. Another challenge that could be proposed would be to expand both the sample size and the number of educational stages involved, including preschool children for the youngest and high school students for the oldest, to provide a comprehensive picture of the evolutionary process of mobility and strength of the spine in children.

## 5 Conclusions

The main findings indicate that global spinal mobility demonstrated weak and inconsistent correlations with trunk strength—absent in three of the four subgroups for the composite strength index and, in secondary school boys, small, negative and no longer significant after adjusting for anthropometric characteristics, except for a persistent negative association with Biering-Sørensen time—which suggests that, in cross-sectional terms, both qualities behave as relatively independent dimensions and should be assessed separately in school-based physical fitness protocols.

These results do not allow the determinants of trunk strength to be identified; factors not assessed in this study, such as neural, coordinative and technical aspects, may play a more relevant role than overall joint range of motion, but this hypothesis requires further investigation. Moreover, owing to the cross-sectional design, no causal or temporal relationship between mobility and strength can be inferred.

However, it can be stated that the protocol is applicable in a single Physical Education session and constitutes a feasible tool for assessing physical function in the school context because of its multicomponent nature, although its accuracy as a screening instrument still needs to be established.

The need for longitudinal studies and educational interventions that combine paravertebral strength training, postural education, and pain monitoring is justified, particularly among pubertal girls and among boys around the growth spurt, in whom the associations observed here were most heterogeneous.

## Data Availability

The raw data supporting the conclusions of this article will be made available by the authors, without undue reservation.
